# Novel Effect of *p*-Coumaric Acid on Hepatic Lipolysis: Inhibition of Hepatic Lipid-Droplets

**DOI:** 10.3390/molecules28124641

**Published:** 2023-06-08

**Authors:** Zhiyi Yuan, Xi Lu, Fan Lei, Hong Sun, Jingfei Jiang, Dongming Xing, Lijun Du

**Affiliations:** 1 College of Pharmacy, Chongqing Medical University, Chongqing 400016, China; yuanzhiyi-06@cqmu.edu.cn; 2 School of Life Sciences and School of Medicine, Tsinghua University, Beijing 100084, China; 3 Institute of Medicinal Plant and Development, Chinese Academy of Medical Sciences and Peking Union Medical College, Beijing 100094, China

**Keywords:** *p*-coumaric acid, hepatic lipase, lipid droplet, NAFLD, PPAR

## Abstract

*p*-coumaric acid (*p*-CA), a common plant phenolic acid with multiple bioactivities, has a lipid-lowering effect. As a dietary polyphenol, its low toxicity, with the advantages of prophylactic and long-term administration, makes it a potential drug for prophylaxis and the treatment of nonalcoholic fatty liver disease (NAFLD). However, the mechanism by which it regulates lipid metabolism is still unclear. In this study, we studied the effect of *p*-CA on the down-regulation of accumulated lipids in vivo and in vitro. *p*-CA increased a number of lipase expressions, including hormone-sensitive lipase (HSL), monoacylglycerol lipase (MGL) and hepatic triglyceride lipase (HTGL), as well as the expression of genes related to fatty acid oxidation, including long-chain fatty acyl-CoA synthetase 1 (ACSL1), carnitine palmitoyltransferase-1 (CPT1), by activating peroxisome proliferator-activated receptor α, and γ (PPARα and γ). Furthermore, *p*-CA promoted adenosine 5′-monophosphate (AMP)-activated protein kinase (AMPK) phosphorylation and enhanced the expression of the mammalian suppressor of Sec4 (MSS4), a critical protein that can inhibit lipid droplet growth. Thus, *p*-CA can decrease lipid accumulation and inhibit lipid droplet fusion, which are correlated with the enhancement of liver lipases and genes related to fatty acid oxidation as an activator of PPARs. Therefore, *p*-CA is capable of regulating lipid metabolism and is a potential therapeutic drug or health care product for hyperlipidemia and fatty liver.

## 1. Introduction

Nonalcoholic fatty liver disease (NAFLD) is the most common chronic liver metabolic syndrome worldwide, associated with obesity and diabetes. The prevalence of NAFLD ranges from 20% to 30% in the general population and up to 75% to 100% in obese individuals. NAFLD starts with simple steatosis and chronic liver inflammation (also called nonalcoholic steatohepatitis, NASH), which in turn can lead to fibrosis, cirrhosis, and hepatocellular carcinoma and increased mortality [1]. Although NAFLD is an important public health problem, no treatment has been especially effective yet [2]. The difficulty of its treatment is that fatty liver manifests mainly as simple steatosis at an early stage and generally does not have symptoms, so the optimal treatment time is easy to miss. Moreover, fatty liver treatment requires long-term medication, which can easily lead to serious adverse reactions, such as hepatotoxicity.

*p*-Coumaric acid (*p*-CA) is known as a supplement of foods, and is a common dietary polyphenol that is present in many natural foods, such as green pepper, carrot, tomato and strawberry. Its major function is as an antioxidant [3,4]. However, increasing research has suggested that it has various effects, including anti-inflammation [5,6], antihyperlipidemia [4,7,8], antihyperglycemia [9,10], antineurodegeneration [11], anticancer [12,13], anticardiac infarction [14] and antibacterial effects [15,16]. Regarding lipid metabolism, *p*-CA was reported to attenuate NAFLD by modulating NOD-like receptor thermal protein domain-associated protein 3 (NLRP3) inflammasome activation and free fatty acid uptake [17,18]. As a common dietary polyphenol in many vegetables and fruits, *p*-CA has the advantages of possessing low toxicity, being easily accepted by patients, and being easy to obtain via plant extraction, chemical synthesis, or microbial fermentation. Therefore, this compound has applicative potential in food health care or in the treatment of fatty liver with drugs, because it is suitable for early preventive and long-term administration. However, its exact mechanism of lipid metabolism remains unknown.

Hepatic lipases participate in lipid metabolism, facilitating serum lipid uptake and degradation, promoting liver lipolysis and preventing liver fat accumulation. Therefore, promoting lipase expression and activity in order to accelerate lipid metabolism is a major concern for treating fatty liver, obesity, and hyperlipidemia. Enzymes for triglyceride metabolism, such as HTGL, MGL, and HSL, play an important role in lipid metabolism [19,20,21]. PPARs (peroxisome proliferator activated receptors) [22,23], a family of nuclear transcription factors, and protein kinase A (PKA) [24,25], a kinase for enzymes that is related to lipid metabolism, play crucial roles in lipid metabolism.

To understand the effect of *p*-CA on lipid metabolism, especially on fatty liver, we conducted studies in vivo and in vitro. Triton WR1339 and oleic acid were used to create a high-lipid model in mouse and HepG2 cells. We measured the expressions of lipases in mouse liver and HepG2 cells and further explored the possible regulatory mechanism of lipase activity. Moreover, lipid droplets were observed in HepG2 cells after being overloaded with oleic acid, and the expression of MSS4, a protein involved in lipid droplet fusion, was also detected.

## 2. Results

### 2.1. p-CA Alleviates Lipid Overloading in Hyperlipidemic Mice and HepG2

**Figure 1:**
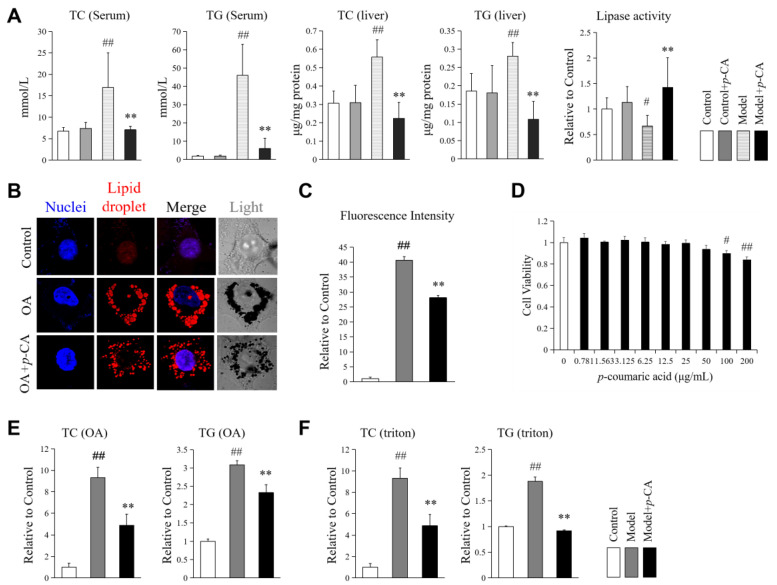
Effect of *p*-coumaric acid (*p*-CA) on the lipid metabolism of mice and HepG2 cells. (**A**): Total cholesterol (TC), total triglycerides (TG) in the blood and liver, and hepatic lipase activity of hyperlipidemic mouse induced by Triton WR1339 (Triton). The hyperlipidemic model was created using Triton WR1339 (300 mg/kg). *p*-CA was administered orally at the dose of 100 mg. (**B**,**C**): Lipid droplets under confocal microscope and the statistical data (*n* = 10–12 cells). The fluorescence intensity represents the integrated fluorescence intensity of oil red O staining per cell. (**D**): Cytotoxicity of *p*-CA in HepG2 cells according to MTT assay. (**E**): TC and TG in cells induced by oleic acid (OA). (**F**): TC and TG in cells induced by Triton RW1339. OA was used at 0.5 mM. Triton was administered at 20 μg/mL. *p*-CA was administered as 10 μg/mL in vitro. The data are presented as the mean ± S.D. from six mice or three independent experiments in vitro. VS. control groups, # *p* < 0.05 and ## *p* < 0.01. VS. hyperlipidemic mice or OA/Triton-treated cells in vitro, ** *p* < 0.01.

Triton WR1339 suppresses the activity of lipoprotein lipases, increases liver acetyl-CoA carboxylase (ACC) activity and up-regulates 3-hydroxy-3-methylglutaryl coenzyme A (HMG-CoA) reductase, resulting in increases in serum and liver concentrations of cholesterol and triglyceride in vivo [26,27,28]. Triton WR1339 is usually used to mimic hyperlipidemic pathological conditions for the screening of lipid-lowering drugs. In our study, *p*-CA reversed the increased total cholesterol (TC) and total triglyceride (TG) induced by Triton WR1339 in mouse serum and liver (Figure 1A). In addition, the activity of total lipases in the liver, which participate in the lipolysis of TC and TG, was detected. Treatment with Triton WR1339 reduced the lipase activity of the mouse liver, while *p*-CA rescued the reduction (Figure 1A).

The same result was observed in vitro. Oleic acid (OA) and Triton WR1339 were used to induce lipid accumulation in HepG2. After oil red O staining, lipid droplets were easily observed under a confocal microscope in oleic-acid-induced cells. In the cells treated with *p*-CA, the lipid droplets were significantly smaller than those in the cells induced by oleic acid (Figure 1B,C). The dose of *p*-CA used in the cell experiments was 10 μg/mL, a safe dosage determined by the methylthiazolyldiphenyl-tetrazolium bromide (MTT) assay, in which the cells were treated for 24 h (Figure 1D). Additionally, the content of TC and TG in the cells, which was elevated by oleic acid and Triton WR1339, was reduced by *p*-CA (Figure 1E,F).

These results confirmed the effect of *p*-CA on reducing intracellular lipid accumulation, and the mechanism may be related to the enhancement of lipase activity.

### 2.2. p-CA Enhances the Expression of MGL HTGL HSL and MSS4 Suppressed by Lipid Overload

**Figure 2:**
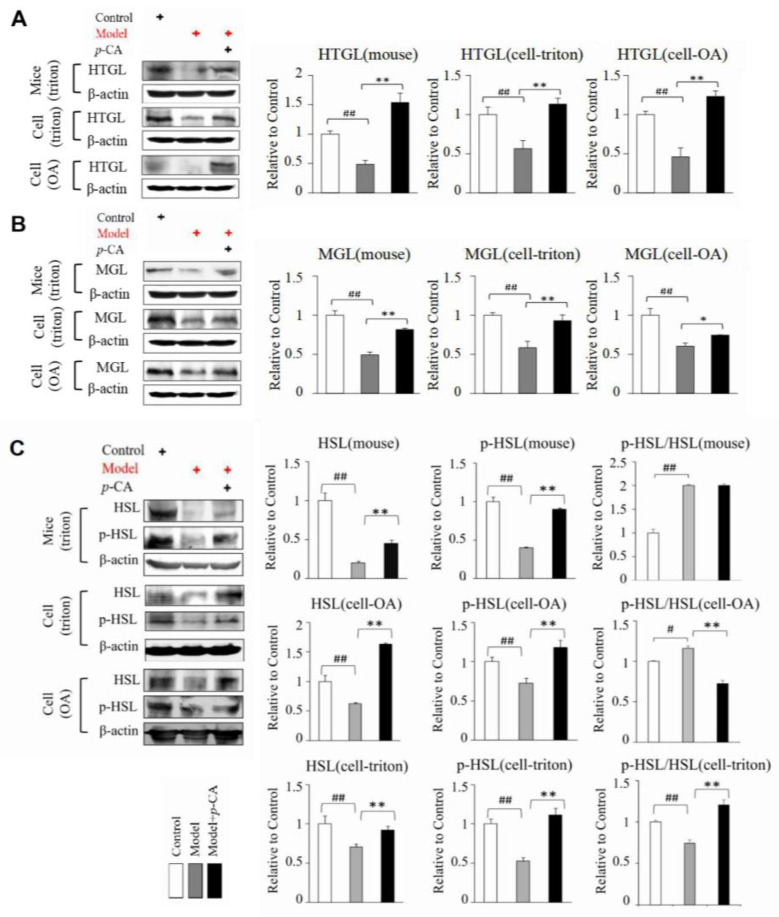
Effect of *p*-CA on the expression of some lipases in mouse liver in vivo and on HepG2 cells in vitro. (**A**): The expression of HTGL. (**B**): The expression of MGL. (**C**): The protein levels of p-HSL and HSL. Triton WR1339 was administered at 300 mg/kg in vivo and 20 μg/mL in HepG2 cells. Oleic acid (OA) was administered at 0.5 mM. The doses of *p*-CA were at 100 mg/kg in mouse model and 10 μg/mL in vitro. The symbol of + in red or black indicates how cells or mice were processed. The data are presented as the mean ± S.D. from six independent mice and three independent experiments in vitro. VS. control, # *p* < 0.05, ## *p* < 0.01. VS. the model mice (cells), * *p* < 0.05, ** *p* < 0.01.

To further investigate whether the lipid-lowering effect of *p*-CA depends on lipolysis regulation, by enhancing lipase activity, we examined the expression of several lipases, namely HSL, HTGL and MGL, in Triton WR1339-induced hyperlipidemic mouse liver and HepG2 cells. The expression level of HTGL and MGL was inhibited in lipid-accumulated tissues and cells, while it increased after *p*-CA administration (Figure 2A,B and Figures S1–S3). Furthermore, *p*-CA also increased the expression of HSL in the mouse and HepG2 cells induced by OA and Triton WR1339 (Figure 2C).

**Figure 3:**
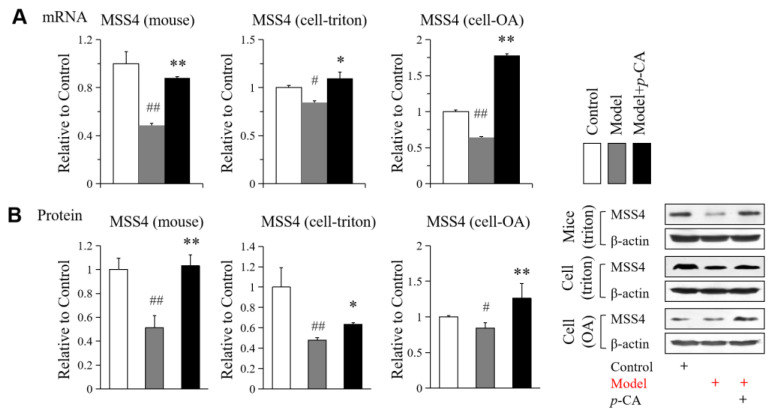
Effect of *p*-CA on the MSS4 in mouse liver in vivo and HepG2 cells in vitro. (**A**): The expression of mRNA. (**B**): The expression of proteins. The symbol of + in red or black indicates how cells or mice were processed. The data are presented as the mean ± S.D. from six independent mice and three independent experiments in vitro. VS. control, # *p* < 0.05, ## *p* < 0.01. VS. the hyperlipidemic mice (cells), * *p* < 0.05, ** *p* < 0.01.

From the previous results in Figure 1B, we also found that *p*-CA was able to reduce the size of lipid droplets induced by oleic acid, accompanied by an increase in the number of miniature fat droplets. To investigate the possible mechanism by which *p*-CA leads to smaller and more lipid droplets, the expression level of MSS4, a negative regulator of lipid droplet growth and fusion [29], was detected. *p*-CA increased MSS4 expression at mRNA and protein levels, while its expression level decreased in hyperlipidemic mice and cells overloaded with lipids (Figure 3A,B).

These results suggested that *p*-CA may enhance lipase activity by increasing the expression of lipases to reduce lipid accumulation, and increase the expression of MSS4 to inhibit lipid droplet fusion and growth.

### 2.3. The Knockdown of MGL HSL and MSS4 Alleviates Lipid-Lowering Effect of p-CA

**Figure 4:**
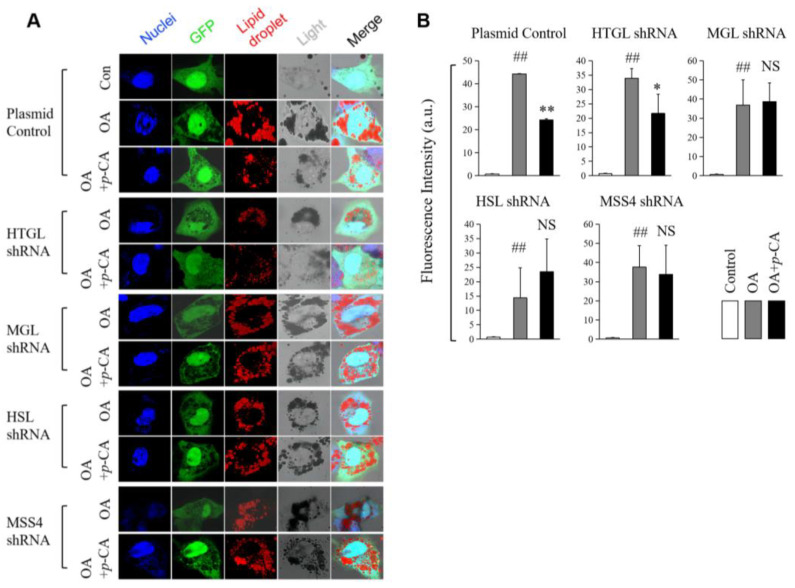
Effect of *p*-coumaric acid (*p*-CA) on lipid droplets in HepG2 cells with the proteins knocked down using shRNA. (**A**): Image of lipid droplets under a confocal microscope. (**B**): The statistical data of integrated fluorescence intensity per cell (*n* = 8–10 cells). The data are presented as the mean ± S.D. from three independent experiments. VS. control, ## *p* < 0.01. VS. OA groups, * *p* < 0.05, ** *p* < 0.01. NS: no significance.

To further confirm the importance of the increased expression of lipases for the lipid-lowering effect of *p*-CA, we knocked down MGL, HTGL and HSL via the transfection of shRNA plasmids. The results showed that the knockdown of MGL and HSL could significantly attenuate the effect of *p*-CA (Figure 4A,B). Thus, we may conclude that the enhanced expression of MGL and HSL was involved in the effect of *p*-CA on lipid overload-suppressed lipolysis. Moreover, when MSS4 was knocked down using shRNA, the effect of *p*-CA on lipid accumulation in cells was also inhibited. In addition, we measured the effect of *p*-CA on the size and number of lipid droplets per cell, when the genes of HTGL, MGL, HSL or MSS4 were knocked down in OA-induced HepG2 cells. The results showed that *p*-CA could reduce the size of lipid droplets and increase the number of small lipid droplets per cell (Figure S4). The knockdown of MSS4 could significantly attenuate the ability of *p*-CA to regulate the size of lipid droplets and the number of lipid droplets in the cells, indicating that the up-regulation of MSS4 is important for decreasing lipid droplet fusion and is involved in the antihyperlipidemic mechanism of *p*-CA. On the other hand, the lipases HTGL, MGL and HSL may participate in the effect of *p*-CA on the morphology of lipid droplets via lipolysis.

### 2.4. Activation of AMPK by p-CA May Participate in Alleviating Lipid Accumulation Independent of HSL Phosphorylation

**Figure 5:**
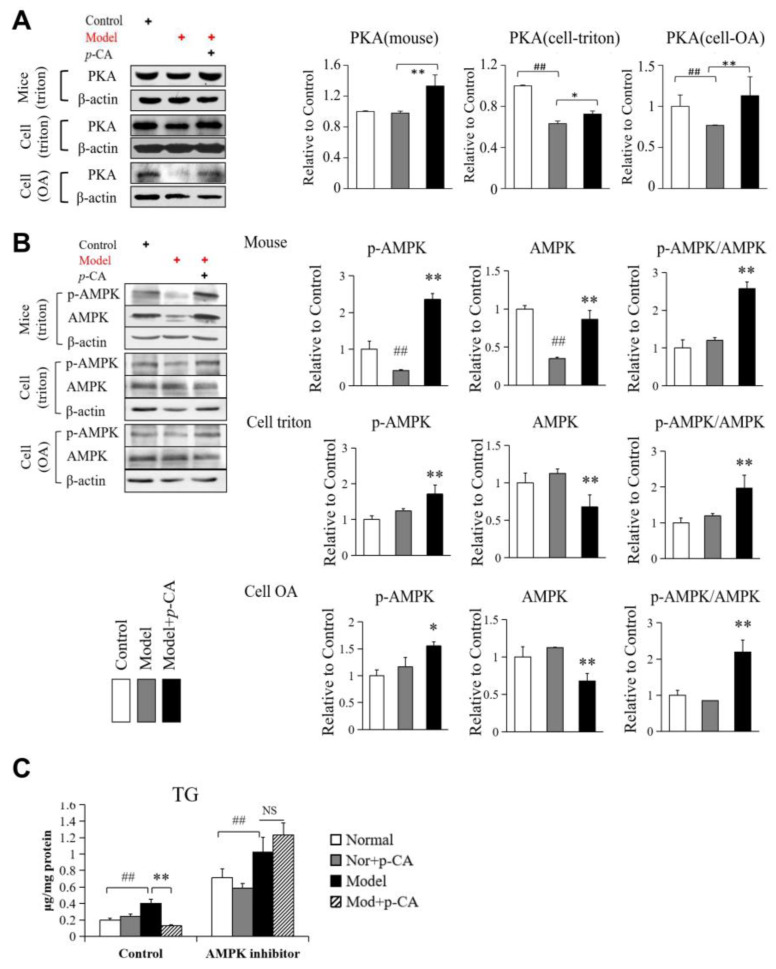
Effect of *p*-CA on the expression of PKA and activation of AMPK in mouse liver in vivo and in HepG2 cells in vitro. (**A**): The expression of PKA. (**B**): The expression and activating phosphorylation of AMPKα at Thr172. (**C**): The effect of *p*-coumaric acid (*p*-CA) after the administration of an AMPK inhibitor, compound C (PubChem CID: 11524144, purchased from Selleck Chemicals, TX, USA). HepG2 cells were pre-treated with 7.5 μM of compound C for 2 h before Triton WR 1339 and *p*-CA. Then, the intracellular TG content was measured. The symbol of + in red or black indicates how cells or mice were processed. The data are presented as the mean ± S.D. from six independent mice and three independent experiments in vitro. VS. control, ## *p* < 0.01. vs. the model mice (cells), * *p* < 0.05, ** *p* < 0.01. NS: no significance.

HSL is an important lipase in liver lipid metabolism and has been proven to be closely related to the lipid-lowering effect of *p*-CA. Unlike MGL and HTGL, the ability of HSL activity to degrade cholesterol and triglycerides is regulated by reversible phosphorylation. The phosphorylation of HSL at Ser563, which stimulates catalytic activity, is triggered by upstream kinases, such as cAMP-dependent protein kinase (PKA). Meanwhile, HSL phosphorylation at Ser565, which inhibits HSL activity, should be mediated by AMP-activated protein kinase (AMPK), which prevents the PKA-dependent phosphorylation and activation of HSL, so that it plays a role in inhibiting lipolysis in adipose tissue [30,31]. Therefore, in order to study the regulation mechanism of *p*-CA on HSL activity, we analyzed the activating phosphorylation level of HSL at Ser563, as well as the activation of PKA and AMPK. The results showed that the level of p-HSL at Ser563 was increased. However, no significant increase was observed in the phosphorylation ratio of HSL when evaluated using p-HSL(Ser563)/HSL (Figure 2C). Furthermore, the expression level of PKA was significantly reduced by lipid overloading, while *p*-CA inhibited its down-regulation (Figure 5A). In addition, *p*-CA also significantly increased the AMPK phosphorylation level, which was evaluated using p-AMPK(Thr172)/AMPK, representing the activation of AMPK (Figure 5B). The increased expression and activity of both PKA and AMPK may be the reason for insignificant changes in the HSL phosphorylation ratio. It suggests that *p*-CA significantly increased the p-HSL level, mainly by elevating the expression of HSL, rather than promoting its phosphorylation.

On the other hand, AMPK is an energy-metabolism-related gene that is reported to be activated by *p*-CA in myocytes and adipocytes [8]. In our study, *p*-CA treatment activated the AMPK pathway by promoting the activating phosphorylation of AMPK at Thr172 instead of its expression (Figure 5B). When AMPK was inhibited by Compound C, an inhibitor of AMPK, the lipid-lowering effect of *p*-CA was antagonized (Figure 5C). The results indicated that the activation of AMPK by *p*-CA may participate in alleviating lipid accumulation independently of HSL phosphorylation.

### 2.5. p-CA Activates PPARα/γ as Transcription Factors Suppressed by Lipid Overload

**Figure 6:**
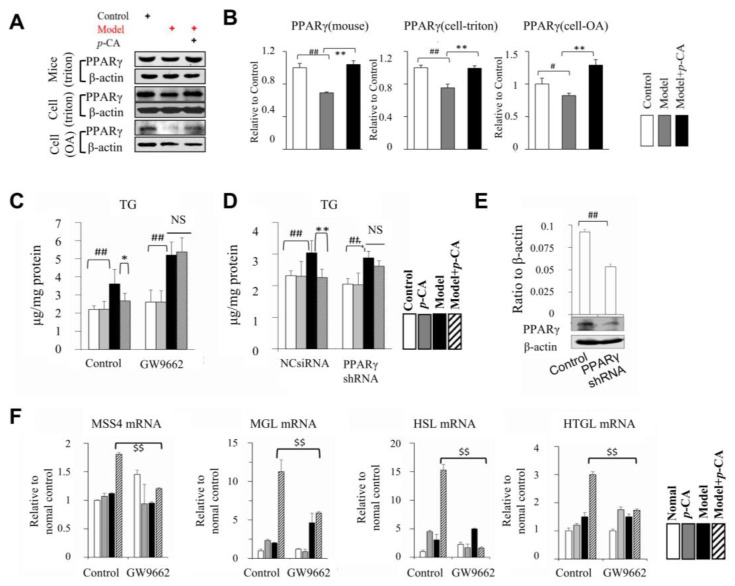
Effect of *p*-CA on the PPARγ in mouse liver in vivo and in HepG2 cells in vitro. (**A**,**B**): The expression level of PPARγ. (**C**): TG in cells induced by Triton WR1339 treated with PPARγ inhibitor, GW9662. (**D**): TG in cells induced by Triton WR1339 when transfected with PPARγ shRNA plasmid. (**E**): The expression of PPARγ when knocked down by shRNA. (**F**): mRNA expression of MSS4, MGL, HSL and HTGL in cells induced by Triton WR1339, treated with or without GW9662. The symbol of + in red or black indicates how cells or mice were processed. The data are presented as the mean ± S.D. from six independent mice and three independent experiments in vitro. VS. the control, # *p* < 0.05, ## *p* < 0.01. VS. the hyperlipidemic mice (cells), * *p* < 0.05, ** *p* < 0.01. VS. the cells treated with Triton WR1339 and *p*-CA, $$ *p* < 0.01. NS: no significance.

To further explore the mechanism of *p*-CA that regulates lipase expression, the peroxisome proliferator-activated receptors (PPARs) were analyzed. PPARγ is an important factor involved in several complicated signaling pathways and plays an essential role in lipid metabolism. At both the mRNA and protein levels, *p*-CA increased the expression of PPARγ (Figure 6A,B). To study its importance regarding the hypolipidemic effects of *p*-CA, a selective antagonist of PPARγ, GW9662 [32], was used in vitro. In control groups, *p*-CA reduced the TG content in the Triton WR1339-induced lipid overloading model. However, when PPARγ was inhibited by GW9662, the lipid-lowering effect of *p*-CA disappeared (Figure 6C). The same result was observed when PPARγ was knocked down by PPARγ shRNA (Figure 6D,E).

**Figure 7:**
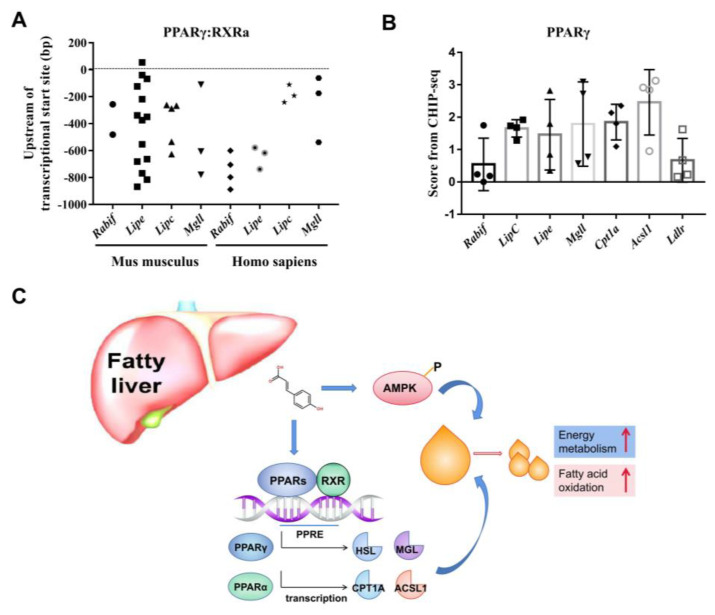
The summary of *p*-CA on lipid metabolism. (**A**): The possible PPARγ-RXRa binding sites, with the promoters of the Rabif, Lipc, Lipe and Mgll genes as transcription factors, were predicted in the eukaryotic promoter database (EPD). These symbols indicated the distance between the predicted binding sites upstream within 1000 bp and the transcription start site in the gene promoter. (**B**): The relationship between PPARγ as a transcription factor and the promoters of the Rabif, Lipc, Lipe, Mgll, Cpt1a and Acsl1 genes was estimated using data from the CHIP-seq in the Cistrome Data Browser (DB). (**C**): The summary of the effect of *p*-CA on lipid metabolism.

Then, we explored whether the activation of PPARγ by *p*-CA was the cause of increased lipase synthesis. When PPARγ was inhibited by GW9662, the effect of *p*-CA on the promotion of HSL, HTGL and MGL expression was significantly inhibited (Figure 6F). The results suggested that PPARγ may be an important factor involved in the effect of *p*-CA on liver lipid metabolism by regulating the expression of HSL, HTGL and MGL. The possible sites of PPARγ–RXRa binding with the promoters of Rabif, Lipe, Lipc and Mgll (correspondingly coding the proteins of MSS4, HSL, HTGL and MGL) as transcription factors were predicted in the eukaryotic promoter database (EPD) (Figure 7A). The results suggested that there were several possible PPARγ–RXRa binding sites in the promoters of Rabif, Lipe, Lipc and Mgll. Then, the relationship between PPARγ and the promoters of Rabif, Lipe, Lipc and Mgll was estimated using data in the samples of HepG2 (GSM2527506), 3T3L1 (GSM1095377 and GSM1571718) and eWAT (GSM2433450) from the chromatin immunoprecipitation sequencing (CHIP-seq) in the Cistrome Data Browser (DB) (Figure 7B). LDLR was chosen as a negative control gene because there were no possible PPARγ–RXRa binding sites on the promoter of LDLR in humans predicated in the EPD, and the mRNA expression of LDLR was not affected by *p*-CA (Figure S5). The mean score of Rabif was close to that of Ldlr and significantly lower than other genes, suggesting that PPARγ was less likely to bind with the promoters of Ldlr and Rabif, while the promoters of other genes may bind with PPARγ as direct target genes. The results of the CHIP-seq assay showed that Lipe, Lipc and Mgll may be the target genes of PPARγ, and that the expression of MSS4 may be indirectly regulated by PPARγ.

Another PPAR, PPARα, and its downstream target genes, including peroxisome carnitine palmitoyltransferase 1a (CPT1A) and acyl-CoA synthetase long-chain family member 1 (ACSL1), which play an important role in fatty acid oxidation, were tested [33,34]. The results showed that *p*-CA was able to promote the expression levels of PPARα, CPT1A and ACSL1 in the hyperlipidemic mouse liver induced by Triton WR1339 and HepG2 cells overloaded with lipids (Figures S6 and S7).

## 3. Discussion

In the present work, we confirm that *p*-CA can decrease TC and TG concentrations in serum and the liver in lipid-overloaded mice and HepG2 cells. This reduction in TC and TG was correlated with the promotion of liver lipase activity and the enhancement of the expression of proteins in lipid metabolism. Moreover, *p*-CA reduced the size of lipid droplets (LDs) in HepG2 cells that were overloaded with oleic acid, suggesting that *p*-CA is a negative regulator of lipid droplet fusion.

HSL is a key enzyme in lipid metabolism [35]. After being phosphorylated by PKA, p-HSL translocates from the cytoplasm towards lipid droplets and acts with phosphorylated perilipin 1 (p-PLIN1) to promote lipolysis in lipid droplets as a lipase [36,37,38]. Our results showed that *p*-CA increased HSL mRNA and protein levels, thereby increasing the level of p-HSL, enhancing its activity and translocation from the cytoplasm to LDs, thus promoting TG degradation in lipid droplets.

In addition to HSL, HTGL and MGL are two crucial lipases in the decomposition of lipids in the liver. HTGL, a lipase mainly expressed in the liver, functions in receptor-mediated lipoprotein uptake, as well as in triglyceride hydrolysis [21,39]. MGL is another essential lipase that contributes to the decomposition of lipid droplets, catalyzing the hydrolysis of monoglycerides [40]. They participate directly in the complete hydrolysis of triglycerides. In this study, *p*-CA up-regulated the expressions of HTGL, HSL and MGL mRNA and protein, enhancing lipid metabolism directly in liver cells and thus agreeing with the increase in total lipase activity. Therefore, HTGL, HSL and MGL are important lipases involved in the mechanism of the lipid-lowering and lipid-droplet fission effects of *p*-CA.

Furthermore, *p*-CA increased the activating phosphorylation level of HSL at Ser563, rather than the phosphorylation ratio. *p*-CA also activated PKA, which participated in the stimulation of the phosphorylation of HSL and significantly elevated the AMPK phosphorylation level as well, preventing the PKA-dependent phosphorylation and activation of HSL. This may explain why *p*-CA has no effect on HSL phosphorylation. Thus, *p*-CA played not only a direct role in lipase expression levels, but also in the activation of AMPK, a classic signaling pathway that regulates lipid metabolism; this consequently reduced the accumulation of fat in the liver, independent of HSL phosphorylation. Therefore, *p*-CA in fatty liver presented a multitarget perspective.

PPARs are ligand-activated transcription factors. The three PPAR isoforms, PPARα, PPARβ/δ (also known as PPARβ or PPARδ) and PPARγ, are found in all mammalian species examined to date. Following ligand binding, PPARs undergo a conformational change that causes the release of histone deacetylase (HDAC) co-repressors, enabling PPARs to heterodimerize with the retinoid X receptor (RXR). RNA polymerase II and coactivators with histone acetyl transferase (HAT) activity are then recruited into this complex, which binds to response elements in target genes, leading to chromatin remodeling and, ultimately, increased transcription.

PPARα, the first PPAR to be identified, is central to maintaining lipid homeostasis. PPARα plays an important role in mobilizing and catabolizing fatty acids, particularly in the liver, where fatty acid oxidation is essential for energy metabolism [41]. PPARα is also the molecular target of drugs that reduce serum lipids by increasing lipid oxidation. The direct target genes of PPARα include enzymes involved in glucose, lipid, and amino acid metabolism, such as CPT1A and ACSL1 [22,42,43]. PPARγ is critical for adipogenesis and fat storage, promoting adipocyte differentiation. White adipose tissue is the primary target of the PPARγ agonists thiazolidinediones (TZDs), which are a common antidiabetic drug used in the clinic. In recent years, the role of PPARγ in the liver has attracted much attention and extensive research. The result of a population-based study showed that the current use of TZDs leads to a 68% reduced risk of NAFLD. In addition to glycemic control, the advantageous effects of TZDs on liver histology include reducing inflammation and redistributing excess triglycerides away from the liver towards adipose tissue [44]. In our study, the results suggested that *p*-CA may play a role similar to PPARα/γ agonists in the regulation of the expression of fatty-acid-catabolizing enzymes and ultimately reduce the accumulation of lipids in the liver. HSL, MGL and HTGL may have been the downstream and direct PPARγ target genes that mediated the lipid-lowering effect of *p*-CA, which is consistent with previous studies [45,46,47]. Meanwhile, CPT1A and ACSL1 were widely recognized as PPARα target genes.

In addition, we observed that the lipid droplets in the cells increased and that MSS4 was up-regulated under oleic-acid-loading conditions. MSS4, which acts as an RAB-interacting factor and negatively regulates Rab8a activity, inhibits the integration and growth of lipid droplets. In MSS4 knockdown 3T3L1 cells, the size of the lipid droplets is significantly increased [29]. Therefore, we measured the size and number of lipid droplets in cells in our study. The results showed that the knockdown of MSS4 could significantly attenuate the effect of *p*-CA on the regulation of the size of lipid droplets and the number of lipid droplets in the cells. This indicated that *p*-CA could up-regulate MSS4 and decrease the fusion of lipid droplets in order to inhibit the accumulation of lipid droplets. This may be a new perspective regarding the effect of *p*-CA on lipid metabolism, in addition to its role in lipolysis and its lipid-lowering effect.

NAFLD has become one of the most common liver diseases in the world. It occurs particularly in patients with central obesity, dyslipidemia, and abnormal glucose tolerance [48]. An early manifestation of fatty liver is the accumulation of neutral fat in the liver and later the formation of lipid droplets in hepatic cells. Therefore, reducing the concentration of lipids and promoting the lipolysis of lipid droplets in the liver are important concerns in preventing and treating NAFLD. In the present work, *p*-CA decreased the TC and TG concentrations in mouse liver and HepG2 cells, promoted lipolysis and the decomposition of lipid droplets, and inhibited the fusion of lipid droplets, suggesting that *p*-CA could have efficacy in the treatment and prevention of NAFLD.

In conclusion, this study showed that *p*-CA lowers serum and liver lipids by up-regulating HSL, HTGL, MGL, CPT1A and ACSL1 by activating PPARα/γ and activating AMPK, and also inhibits the fusion and growth of lipid droplets by increasing the MSS4 expression level (Figure 7C). Because many plants and natural foods contain *p*-CA, the results of the present study could form a basis for the rational use of these natural foods in order to prevent fatty liver and obesity, and have important theoretical significance for understanding the mechanism of *p*-CA in lipid metabolism.

Furthermore, intracellular lipids with different subcellular localizations display different distributions, dynamics and function. The nuclear lipids, including phosphatidylinositol phosphates (PIPs), have been reported to play an important role in gene expression regulation [49,50]. In our research, *p*-CA was verified to play an important role in the promotion of lipolysis by activating PPARs, but whether it is involved in regulating gene expression by affecting nuclear lipids remains unclear; this is worth in-depth study. Some super-resolution imaging methods, such as single-molecule localization microscopy (SMLM), stochastic optical reconstruction microscopy (STORM) and so on, provide an approach for the quantitative evaluation of the sub-nuclear distribution of nuclear lipids with nanometer resolution and millisecond precision [51,52]. This will greatly benefit the development of nuclear lipid biology.

## 4. Materials and Methods

### 4.1. Experimental Animals, Drugs and Chemicals

Male ICR mice weighing 18–22 g were purchased from Vital River Laboratories (Beijing, China) and housed in temperature (25 ± 1 °C) and humidity-controlled (50%) rooms, kept on a 12 h light/dark cycle and provided with rodent chow and drinkable water ad libitum. The laboratory animal facility has been accredited by the Association for Assessment and Accreditation of Laboratory Animal Care International, and the Institutional Animal Care and Use Committee (IACUC) of Tsinghua University approved all animal protocols used in this study (Approval ID: 14-DLJ-CA). *p*-CA (PubChem CID: 637542, purity > 98%) and Triton WR1339 (Triton) were purchased from Sigma (St. Louis, MO, USA). Oleic acid (OA) was purchased from Beijing Chemical Plant (Beijing, China). Kits for TC (cholesterol) and TG (Triglycerides) were purchased from Zhongsheng Beikong Biochemical Co., Ltd. (Beijing, China). Kits for lipase activity and protein were purchased from Nanjing Jiancheng Bioengineering Institute (Nanjing, China) and Beyotime Biotechnology (Shanghai, China). Human HepG2 cells (a human hepatocyte carcinoma cell line) were provided by the Center of Cells, Chinese Academy of Medical Sciences (Beijing, China).

### 4.2. Hyperlipidemic Model in Mice Induced by Triton WR1339

The mice were divided into three groups: a normal control, a hyperlipidemia model and a *p*-CA group. The control was treated with normal saline, while the other two groups were treated with Triton WR1339 to induce the hyperlipidemic model in mice. Triton WR1339 was dissolved in normal saline and injected into the circulation of mice as a single, intravenous administration through the mouse tail vein at a dose of 350 mg/kg. Then, the mice were deprived of food for 24 h. In the drug groups, *p*-CA was given by intragastric administration at the dose of 100 mg/kg at the same time. Then, 24 h after Triton WR1339 treatment, blood was taken from the supraorbital sinus to measure the total cholesterol (TC) and total triglyceride (TG), and the livers were immediately removed and kept at −80 °C for RNA, protein, TC and TG measurement.

### 4.3. Cell Culture and Cell Viability Assay

HepG2 cells were maintained in DMEM (Gibco, Carlsbad, CA, USA) containing 10% fetal bovine serum (FBS) (Sijiqing Biotech, Beijing, China) in 5% CO_2_ at 37 °C. The cytotoxicity of *p*-CA in HepG2 cells treated for 24 h was evaluated via MTT assay.

### 4.4. The Triton WR1339 and Oleic Acid-Induced Hyperlipidemic Model in HepG2 Cells

HepG2 cells were seeded into 6-well plates. The medium was changed to medium containing 0.5 mM of oleic acid (OA) or 20 μg/mL of Triton WR1339 diluted in DMEM in order to induce intracellular lipid accumulation. In the drug group, *p*-CA (10 μg/mL) was added at the same time. After treatment with OA or Triton WR1339, with or without *p*-CA, for 24 h, cells were harvested for TC and TG measurement and mRNA and protein expression.

### 4.5. The Measurement of TC and TG Content

HepG2 cells were harvested by centrifugation at 800× *g* for 5 min and washed with phosphate-buffered saline (PBS) 3 times. The cell pellet was suspended in 100 μL of 50% ethanol and disrupted by repetitive freeze–thawing. After centrifugation at 11,000× *g* for 15 min at 4 °C, the supernatant was measured by kits for TC and TG according to the manufacturer’s instructions. Protein was also measured by a protein kit for normalization. The sera of mice were isolated via centrifugation at 5000× *g* for 5 min. TC and TG were also tested using TC and TG kits. One hundred milligrams of liver tissue was weighed, chopped and ground with 1 mL of ice-cold PBS. After centrifugation at 11,000× *g* for 15 min at 4 °C, the TC, TG and protein of the resulting supernatant were measured using kits.

### 4.6. The Measurement of Lipase Activity

The liver tissue of mice (100 mg) was sufficiently disrupted in 1 mL of ice-cold PBS and centrifuged at 1500× *g* for 10 min at 4 °C. The resulting supernatant was tested using a hepatic lipase assay kit (Jiancheng Bioengineering Institute, Nanjing, China) according to the protocol of the kit. The reagents were added into the samples and mixed. Then, the samples were placed in the water bath (37 °C) for 30 min and were tested at 550 nm. The content of protein was determined using a protein reagent kit (BioSino Bio-Technology and Science Inc., Beijing, China). Data were expressed as U/mg protein.

### 4.7. Quantitative PCR and Western Blotting

Quantitative PCR (qPCR) was carried out as previously described [53]. All the primers used in these analyses were synthetized in Sangon Biotechnology Ltd. (Shanghai, China). Their sequences are shown in Table 1.

Western blot analysis was performed as previously described [53]. β-Actin was employed as an internal reference. The primer antibodies for HTGL, MGL, HSL, p-HSL, AMPK, p-AMPK and PPARα were purchased from Bioworld (Shanghai, China). The primary antibodies for ACSL1, AOX and CPT1A were all purchased from ABclonal (Wuhan, China). Primary antibodies for β-actin and MSS4, as well as HRP-conjugated goat anti-rabbit and rabbit anti-mouse IgG, were purchased from Santa Cruz (Dallas, TX, USA).

### 4.8. Lipid Droplet Observed by a Confocal Microscopy

HepG2 cells were seeded into perforated plates (Corning, NY, USA) and treated with oleic acid and *p*-CA for 24 h. Cells were fixed with 4% paraformaldehyde for 15 min. Then, 0.5 g of oil red O powder was dissolved in 100 mL of isopropanol (stored at 4 °C), diluted by H_2_O (3:2) and filtered using qualitative filter paper to remove impurities. Next, cells were stained with oil red O for 10 min, followed by Hoechst 33,342 (5 μg/mL) for 5 min. The lipid droplets stained by oil red O and the nuclei stained by Hoechst 33,342 were photographed using a Zeiss LSM 710 (Carl Zeiss, Germany).

### 4.9. Construction of MSS4, HSL, MGL, HTGL and PPARγ shRNAs

The plasmid of shRNA used for knockdown was pLL3.7. The sequences of MSS4, HSL, MGL, HTGL and PPARγ shRNA are shown in Table 2. They were annealed to double strands and then inserted into a pLL3.7 vector dealt with HpaI and XhoI. The shRNA plasmids were transfected into HepG2 by PEI to generate knockdown cells. Then, 24 h later, the MSS4, HSL, MGL and HTGL knockdown cells were treated with oleic acid and *p*-CA, stained with oil red and then observed using a confocal microscope.

### 4.10. Data Analysis

All data are expressed as the mean ± S.D. Data were statistically analyzed using the Kruskal–Wallis test in R software version 4.2.3 (Indianapolis, IN, USA). *p* < 0.05 was considered statistically significant.

## Figures and Tables

**Table 1:** Sequences of primers used in qPCR.

Primers(bp)	Sequences	Primers(bp)	Sequences
H-MSS4(154) NM_002871.4	CTTTTCCTTCCCTCCATGAGAA	M-MSS4(184) NM_145510.1	CAGCAATCCTGATGGTGATG
	AGACCAGAAACTTGATGTTGCCCA		CTCCAAGGCCACATAGAAGC
H-HSL(144) NM_005357.3	CTCCTCCTATTCCTAATCCTCC	M-HSL(106) NM_010719.5	CCAGCCTGAGGGCTTACTG
	CACTTCCTCTTGGGTTTCACTC		CTCCATTGACTGTGACATCTCG
H-PKA(198) NM_002730.3	GCGTGAAAGAATTCTTAGCCA	M-PKA(119) NM_008854.5	AGATCGTCCTGACCTTTGAGT
	CCACCTTCTGTTTGTCGAGGA		GGCAAAACCGAAGTCTGTCAC
H-HTGL(276) NM_000236.2	CTGGTGGTGACATGAACAGC	M-HTGL(207) NM_008280.2	ATGGGAAATCCCCTCCAAATCT
	CTTTGCCCAGAGTGATGGGA		GTGCTGAGGTCTGAGACGA
H-MGL(411) NM_007283.6	CAATCCTGAATCTGCAACAACTTTC	M-MGL(127) NM_011844.4	CGGACTTCCAAGTTTTTGTCAGA
	ATGTTTATTTCATGGAAGACGGAGT		GCAGCCACTAGGATGGAGATG
H-PPARγ(272) NM_015869.4	CTGCGAAAGCCTTTTGGTGACTTTATGG	M-PPARγ(272) NM_011146.3	CTGCGGAAGCCCTTTGGTGACTTTATGG
	ACAATCTGTCTGAGGTCTGTCATTTTCT		ACGATCTGCCTGAGGTCTGTCATCTTCT
M-ACSl1(119) NM_007981.4	TCTTCCCTGTGGTTCCCAG	M-PPARα(76) NM_011144.6	GACAAGGCCTCAGGGTACCA
	AAGCTCCGCCTCTTTCCTTT		GCCGAATAGTTCGCCGAAA
M-AOX1(270) NM_009676.2	CAAAGATGGAGAACGGCG	M-CPT1A(84) NM_013495.2	CCTGGGCATGATTGCAAAG
	GCACTGATGATAGTTGAACCG		ACGCCACTCACGATGTTCTTC
M-AMPKα1(91) NM_001013367.3	CTACCTAGCAACCAGCCCAC	M-AMPKα2(75) NM_178143.2	GAAAGGATGCCGCCTCTCAT
	TTCGGCAACCAAGAACGGTA		GGGCTTCGTTGTGTTGAGTG

Note: H: human. M: mouse. The NM number is the acronym used by the National Center for Biotechnology Information (NCBI) for identifying genes in biological specimens.

**Table 2:** Sequences of shRNAs.

Gene Name		Sequences
HSL	F	TCCCTCCGATGTCAACTTCTTATTCAAGAGATAAGAAGTTGACATCGGAGGGTTTTTTC
	R	TCGAGAAAAAACCCTCCGATGTCAACTTCTTATCTCTTGAATAAGAAGTTGACATCGGAGGGA
MGL	F	TCCAATCCTGAATCTGCAACAATTCAAGAGATTGTTGCAGATTCAGGATTGGTTTTTTC
	R	TCGAGAAAAAACCAATCCTGAATCTGCAACAATCTCTTGAATTGTTGCAGATTCAGGATTGGA
HTGL	F	TGCATGAGATGAAGACCAGATTTTCAAGAGAAATCTGGTCTTCATCTCATGCTTTTTTC
	R	TCGAGAAAAAAGCATGAGATGAAGACCAGATTTCTCTTGAAAATCTGGTCTTCATCTCATGCA
MSS4	F	TACGTGGGCAACATCAAGTTTCTTCAAGAGAGAAACTTGATGTTGCCCACGTTTTTTTC
	R	TCGAGAAAAAAACGTGGGCAACATCAAGTTTCTCTCTTGAAGAAACTTGATGTTGCCCACGTA
PPARγ	F	TGACAACAGACAAATCACCATTTTCAAGAGAAATGGTGATTTGTCTGTTGTCTTTTTTC
	R	TCGAGAAAAAAGACAACAGACAAATCACCATTTCTCTTGAAAATGGTGATTTGTCTGTTGTCA

## Data Availability

All the data or resources are available in the Source Data file or from the corresponding author upon reasonable request.

## Supplementary Materials

The following supporting information can be downloaded at: https://www.mdpi.com/article/10.3390/molecules28124641/s1. Figure S1. Effect of *p*-coumaric acid (*p*-CA) on mRNA expression of the proteins involved in lipid metabolism. (A): The expression of mouse liver (n = 6). (B): The expression of HepG2 cells induced with Triton WR1339. (C): The expression of HepG2 cells induced with OA. The data are presented as the mean ± S.D. from six independent mice and three independent experiments in vitro. Figure S2. Effect of *p*-coumaric acid (*p*-CA) on mRNA expression of the proteins involved in lipid metabolism in normal mouse liver. (A): The expression of mRNA. (B): The expression of protein. The data are presented as the mean ± S.D. from three independent experiments. There were no statistical significance between the control and *p*-CA groups. Figure S3. Effect of *p*-coumaric acid (*p*-CA) on mRNA expression of the proteins involved in lipid metabolism in normal HepG2 cells. (A): The expression of mRNA. (B): The expression of protein. The data are presented as the mean ± S.D. from three independent experiments. There were no statistical significance between the control and *p*-CA groups. Figure S4. Effect of *p*-coumaric acid (*p*-CA) on the size of lipid droplets and the number per cell, when the gene of HTGL, MGL, HSL or MSS4 was knocked down in OA induced HepG2 cells. (A): The size (area) of lipid droplets (n = 300–500 lipid droplets). (B): The number of lipid droplets per cell (n = 8–10 cells). The symbol * in (B) is vs the OA group in the negative control of plasmid PLL3.7. The data are from three independent experiments. vs. the OA group, * *p* < 0.05. ** *p* < 0.01, ** *p* < 0.01, **** *p* < 0.0001. NS: no significance. Figure S5. mRNA expression of LDLR after administration of *p*-coumaric acid (*p*-CA) in mouse liver and HepG2 cells. Data are presented as the mean ± S.D. from six mice or three independent experiments in vitro. Figure S6. mRNA expression of PPARα, CPT1A, ACSL1, AMPK and AOX1 after administration of *p*-coumaric acid (*p*-CA) in mouse liver. Triton WR1339 was administered at 350 mg/kg in mouse. The doses of *p*-CA were at 100 mg/kg. Data are presented as the mean ± S.D. from six mice (three independent experiments). VS. the control, # *p* < 0.05, ## *p* < 0.01. **, VS. the hyperlipidemic mice, * *p* < 0.05, ** *p* < 0.01. NS: no significance. Figure S7. Effect of *p*-CA on the PPARα and its downstream target genes CPT1A and ACSL1 in mouse liver in vivo and HepG2 cells in vitro. (A): The expression of PPARα. (B): The expression of CPT1A. (C): The expression of ACSL. The concentration of Triton WR1339, OA and *p*-CA used was the same as that in Figure 2. The symbol of + in red or black indicates how cells or mice were processed. The data are presented as the mean ± S.D. from six independent mice and three independent experiments in vitro. VS. the control (normal), # *p* < 0.01, ## *p* < 0.01. VS. the model mice (cells), * *p* < 0.05 and ** *p* < 0.01.

## Sample Availability

Sample of the compound *p*-CA is available from the authors.

## Abbreviations

*p*-CA, *p*-coumaric acid; TC, total cholesterol; TG, total triglycerides; OA, oleic acid; HSL, hormone-sensitive lipase; PLIN1, perilipin 1; HTGL, hepatic triglyceride lipase; MGL, monoacylglycerol lipase; MSS4, mammalian suppressor of Sec4; NAFLD, non-alcoholic fatty liver disease; ACSL, long chain fatty acyl-CoA synthetase; CPT1, carnitine palmitoyltransferase-1; PPARα, peroxisome proliferator-activated receptor α; AMPK, AMP-activated protein kinase; p-AMPK, phosphorylation of AMP-activated protein kinase
