# Supplementary material for: Novel Effect of p-Coumaric Acid on Hepatic Lipolysis: Inhibition of Hepatic Lipid-Droplets
Source: Molecules. 2023 Jun 8;28(12):4641. doi: 10.3390/molecules28124641 (PMC10303986; doi:10.3390/molecules28124641)
Supplement: Supplementary file 1 [file molecules-28-04641-s001.zip › molecules-2412173-supplementary.pdf]

Article

Novel effect of *p*-coumaric acid on hepatic lipolysis: Inhibition of hepatic lipid-droplets

Zhiyi Yuan[1], Xi Lu[2], Fan Lei[2], Hong Sun[3], Jingfei Jiang[2], Dongming Xing[2], Lijun Du[2,*]

[1] College of Pharmacy, Chongqing Medical University, Chongqing 400016, China; yuanzhiyi-06@cqmu.edu.cn
[2] School of Life Sciences and School of Medicine, Tsinghua University, Beijing 100084, China
[3] Institute of Medicinal Plant and Development, Chinese Academy of Medical Sciences and Peking Union Medical College, Beijing 100094, China
* Correspondence: lijundu@mail.tsinghua.edu.cn

Supplementary Materials

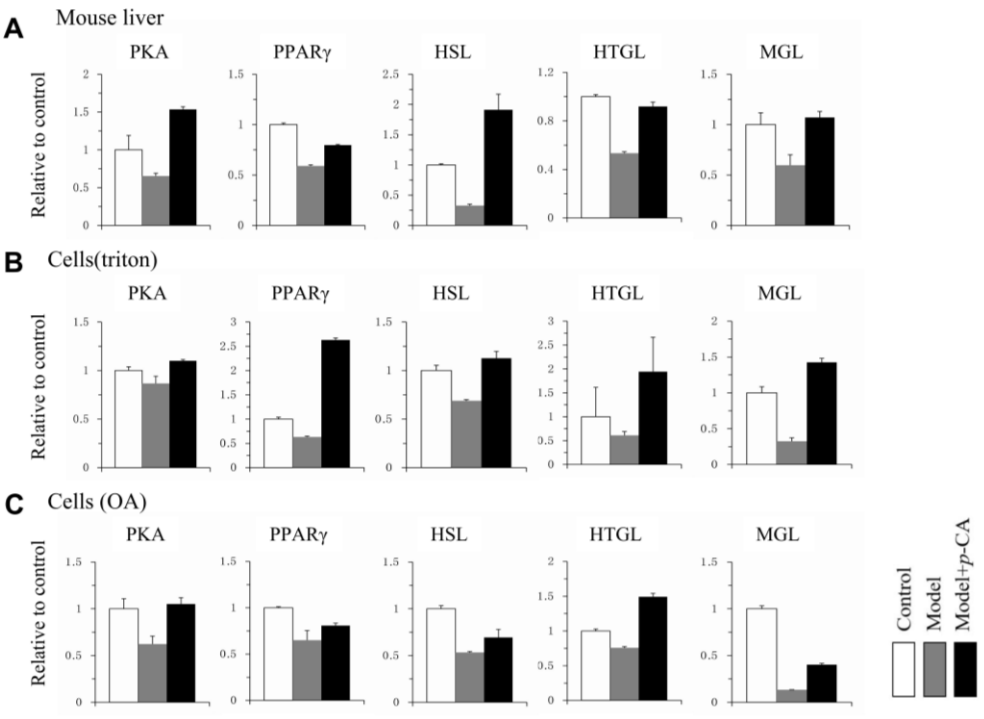

Figure S1. Effect of *p*-coumaric acid (*p*-CA) on mRNA expression of the proteins involved in lipid metabolism. (A): The expression of mouse liver (*n*=6). (B): The expression of HepG2 cells induced with Triton WR1339. (C): The expression of HepG2 cells induced with OA. The data are presented as the mean ± S.D. from six independent mice and three independent experiments *in vitro*.

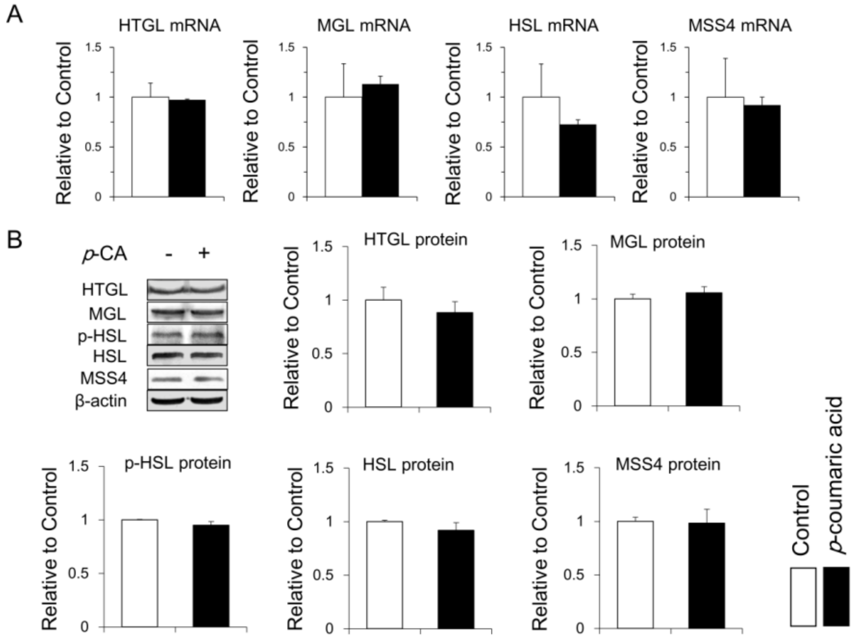

Figure S2. Effect of *p*-coumaric acid (*p*-CA) on mRNA expression of the proteins involved in lipid metabolism in normal mouse liver. (A): The expression of mRNA. (B): The expression of protein. The data are presented as the mean ± S.D. from three independent experiments. There were no statistical significance between the control and *p*-CA groups.

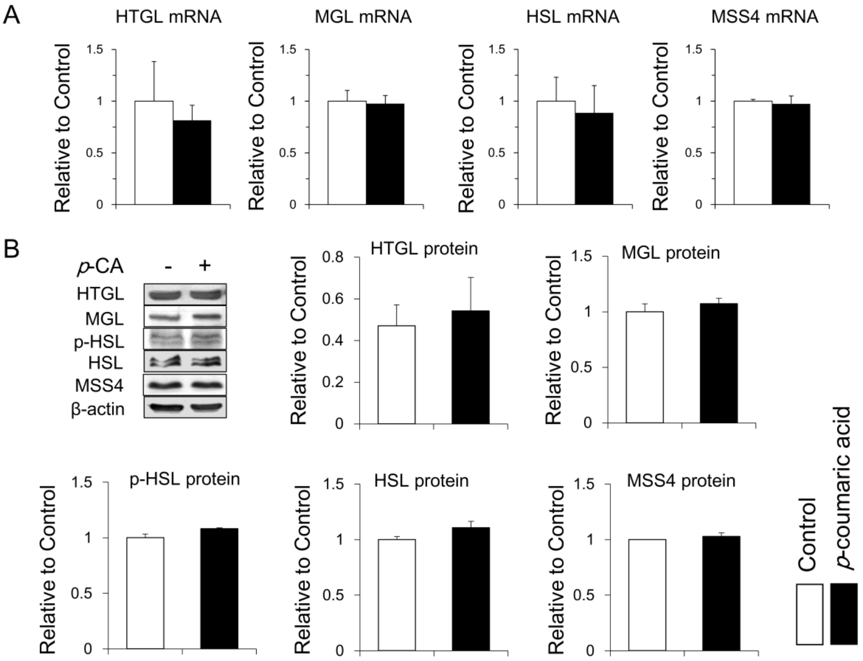

Figure S3. Effect of *p*-coumaric acid (*p*-CA) on mRNA expression of the proteins involved in lipid metabolism in normal HepG2 cells. (A): The expression of mRNA. (B): The expression of protein. The data are presented as the mean ± S.D. from three independent experiments. There were no statistical significance between the control and *p*-CA groups.

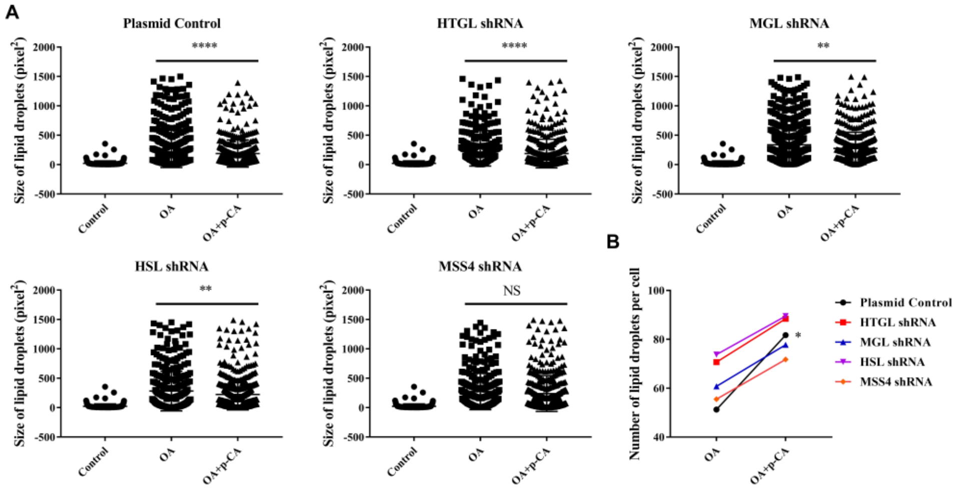

Figure S4. Effect of *p*-coumaric acid (*p*-CA) on the size of lipid droplets and the number per cell, when the gene of HTGL, MGL, HSL or MSS4 was knocked down in OA induced HepG2 cells. (A): The size (area) of lipid droplets (*n*=300-500 lipid droplets) . (B): The number of lipid droplets per cell (*n*= 8-10 cells). The symbol * in (B) is vs the OA group in the negative control of plasmid PLL3.7. The data are from three independent experiments. vs. the OA group, *$P < 0.05$. **$P < 0.01$, **$P < 0.01$, ****$P < 0.0001$. NS: no significance.

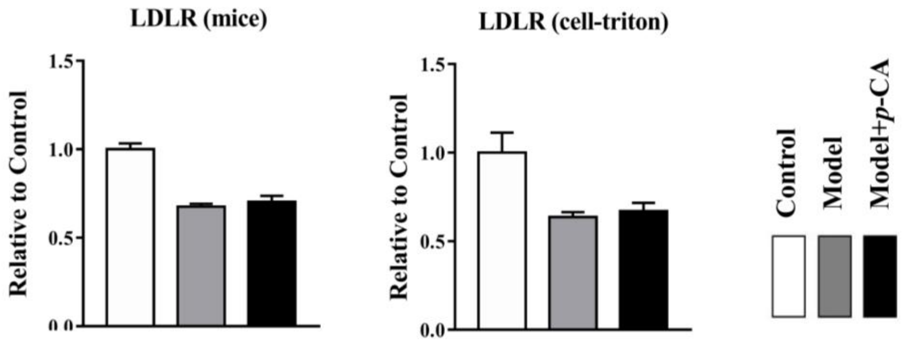

Figure S5. mRNA expression of LDLR after administration of *p*-coumaric acid (*p*-CA) in mouse liver and HepG2 cells. Data are presented as the mean ± S.D. from six mice or three independent experiments *in vitro*.

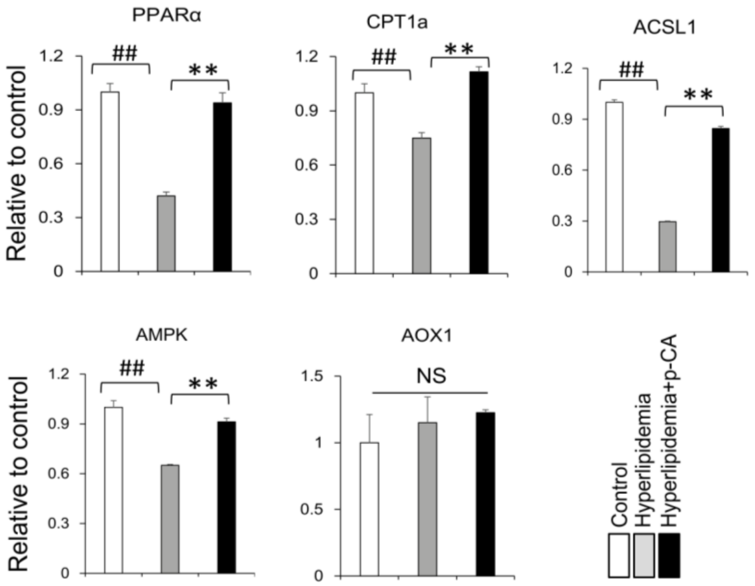

Figure S6. mRNA expression of PPAR*α*, CPT1A, ACSL1, AMPK and AOX1 after administration of *p*-coumaric acid (*p*-CA) in mouse liver. Triton WR1339 was administered at 350 mg/kg in mouse. The doses of *p*-CA were at 100 mg/kg. Data are presented as the mean ± S.D. from six mice (three independent experiments). VS. the control, $^{\#}P < 0.05$, $^{\#\#}P < 0.01$. **, VS. the hyperlipidemic mice, $^{*}P < 0.05$, $^{**}P < 0.01$. NS: no significance.

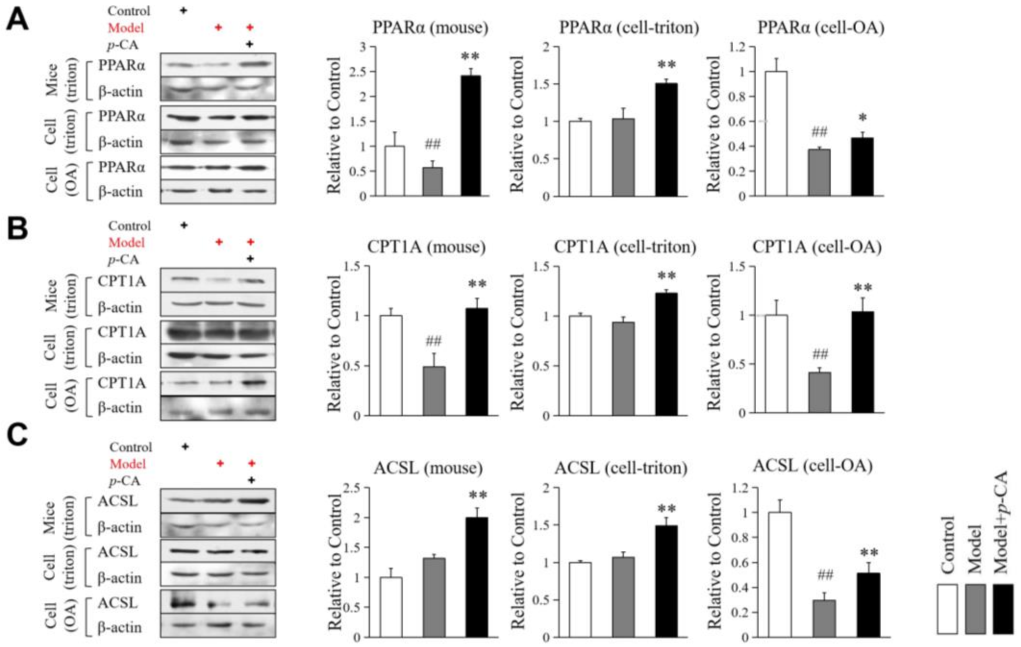

Figure S7. Effect of *p*-CA on the PPAR*α* and its downstream target genes CPT1A and ACSL1 in mouse liver *in vivo* and HepG2 cells *in vitro*. (A): The expression of PPAR*α*. (B): The expression of CPT1A. (C): The expression of ACSL. The concentration of Triton WR1339, OA and *p*-CA used was the same as that in Fig.2. The symbol of + in red or black indicates how cells or mice were processed. The data are presented as the mean ± S.D. from six independent mice and three independent experiments *in vitro*. *VS.* the control (normal), $^{\#}P < 0.01$, $^{\#\#}P < 0.01$. *VS.* the model mice (cells), $^{*}P < 0.05$ and $^{**}P < 0.01$.
